# Bacterial Communities Vary between Sinuses in Chronic Rhinosinusitis Patients

**DOI:** 10.3389/fmicb.2015.01532

**Published:** 2016-01-22

**Authors:** Tom V. Joss, Catherine M. Burke, Bernard J. Hudson, Aaron E. Darling, Martin Forer, Dagmar G. Alber, Ian G. Charles, Nicholas W. Stow

**Affiliations:** ^1^Faculty of Medicine, Sydney Medical School, University of SydneySydney, NSW, Australia; ^2^Faculty of Science, The Ithree Institute, University of TechnologySydney, NSW, Australia; ^3^Department of Microbiology and Infectious Disease, Royal North Shore HospitalSydney, NSW, Australia; ^4^Department of Otolaryngology, Royal North Shore HospitalSydney, NSW, Australia; ^5^Department of Otolaryngology, Mona Vale HospitalSydney, NSW, Australia

**Keywords:** chronic rhinosinusitis, microbiome, sinus, 16S rRNA gene sequencing, swabs

## Abstract

Chronic rhinosinusitis (CRS) is a common and potentially debilitating disease characterized by inflammation of the sinus mucosa for longer than 12 weeks. Bacterial colonization of the sinuses and its role in the pathogenesis of this disease is an ongoing area of research. Recent advances in culture-independent molecular techniques for bacterial identification have the potential to provide a more accurate and complete assessment of the sinus microbiome, however there is little concordance in results between studies, possibly due to differences in the sampling location and techniques. This study aimed to determine whether the microbial communities from one sinus could be considered representative of all sinuses, and examine differences between two commonly used methods for sample collection, swabs, and tissue biopsies. High-throughput DNA sequencing of the bacterial 16S rRNA gene was applied to both swab and tissue samples from multiple sinuses of 19 patients undergoing surgery for treatment of CRS. Results from swabs and tissue biopsies showed a high degree of similarity, indicating that swabbing is sufficient to recover the microbial community from the sinuses. Microbial communities from different sinuses within individual patients differed to varying degrees, demonstrating that it is possible for distinct microbiomes to exist simultaneously in different sinuses of the same patient. The sequencing results correlated well with culture-based pathogen identification conducted in parallel, although the culturing missed many species detected by sequencing. This finding has implications for future research into the sinus microbiome, which should take this heterogeneity into account by sampling patients from more than one sinus.

## Introduction

Chronic rhinosinusitis (CRS) is defined as inflammation of the nasal cavity and paranasal sinus mucosa for more than 12 weeks, while nasal polyposis (NP) is considered a subgroup of CRS, where polyps appear in the nasal cavity (Fokkens et al., 2012). Symptoms of CRS include nasal blockage and discharge, facial pain and discomfort, and reduction or loss of smell. These symptoms can have a significant impact on quality of life, particularly on social functioning and bodily pain scores (Metson and Gliklich, 2000). The high lifetime prevalence of 14–16% means that CRS also has a significant impact on society in terms of direct and indirect healthcare costs, as well as lost work days (Bhattacharyya, 2009). Choosing the most effective treatment strategy for CRS can be challenging, in part due to our lack of understanding of its pathogenesis.

The pathogenesis of CRS is complex, and involves an interaction between multiple factors such as microbial infection, immune dysfunction, and mucociliary impairment (Hamilos, 2014). The relative importance and precedence of these factors is a subject of continuing debate; some postulate that immune dysfunction leads to inflammation and blockage of the sinus, which then influences the microbial flora (Ooi et al., 2008), while others argue that changes in the microbial flora initiate the inflammatory response (Ou et al., 2014). Studies of sinus microbes have traditionally relied on culture-based methods, but more recently researchers have begun using culture-independent molecular techniques (Stephenson et al., 2010; Stressmann et al., 2011; Abreu et al., 2012; Feazel et al., 2012; Aurora et al., 2013; Boase et al., 2013). These techniques are able to provide a more complete survey of the microbes found in the sinuses of people with CRS, and offer the potential to find particular species that may be implicated in the pathogenesis of CRS.

Culture-independent molecular techniques that have been used to investigate the microbiome of CRS include pyrosequencing and DNA microarray analysis of the 16S rRNA gene, fluorescence *in situ* hybridization and mass spectrometry. A variety of sampling methods have also been used, including taking swabs, lavages, sinus brushings, or tissue biopsies from various sites (Table 1). The results of these studies are even more variable than the methods used, and there are many conflicts. Different studies have identified different bacteria as being more abundant in CRS than in controls, such as *Pseudomonas aeruginosa* (Stressmann et al., 2011), *Corynebacterium tuberculostearicum* (Abreu et al., 2012), and Cyanobacteria (Aurora et al., 2013). Stephenson et al. (2010) found that the sinus microbiome of CRS patients was predominantly composed of the anaerobic bacteria *Propionibacterium, Diaphorobacter*, and *Peptoniphilus*, while control subjects had higher frequencies of *Staphylococcus* and *Corynebacterium*. Feazel et al. (2012) reported that *Staphylococcus, Corynebacterium*, and *Propionibacterium* were the most frequently identified bacteria in CRS patients, while Boase et al. (2013) found that *Staphylococcus aureus* was more common, and *Propionibacterium* was less common among CRS patients. The variability between studies could be explained by sample population differences, as well as differences in sample collection and DNA extraction methods, which have been demonstrated to influence the relative abundances of bacterial taxa detected by sequencing (Morgan et al., 2010). Additionally, all of these studies relied on analyzing a single sample from each patient, working on the assumption that this one sample would be representative of the entire sinus microbiome for that CRS patient. Previous studies of different environments within the nasal cavity have identified differing microbial populations (Yan et al., 2013; Biswas et al., 2015), but as yet, no one has determined whether microbial communities differ between sinuses within an individual.

**Table 1:** **Summary of culture-independent research methods used to study the microbiome of CRS**.

**Citation**	**Sample type**	**Sample site**	**Molecular technique**
Stephenson et al., 2010	Swab	Site of maximal purulence or surface of maxillary sinus	Pyrosequencing of 16S gene
Stressmann et al., 2011	Tissue and mucin	Polyp and inferior turbinate	T-RFLP of 16S gene
Feazel et al., 2012	Swab	Area of mucopurulence or the middle meatus	Pyrosequencing of 16S gene
Abreu et al., 2012	Brushings	Lateral, central, and medial portions of the maxillary sinus	DNA microarray analysis of 16S gene
Boase et al., 2013	Tissue	Ethmoid sinus	PCR and mass spectrometry; fluorescence *in situ* hybridization
Aurora et al., 2013	Lavage	Superior middle meatus	Pyrosequencing of 16S and 18S genes

The primary objective of this study was to test the hypothesis that bacterial communities are the same in the different sinuses of an individual with CRS. Secondary objectives were to determine whether sampling with swabs generates equivalent data to tissue sampling, and whether swabbing the nostril generates equivalent data to swabbing the sinuses, which require surgery to access. We found that variation does exist in different sinuses of the patients, and in some case this variation is substantial. The potential implications of this variation are discussed.

## Materials and methods

### Patient recruitment

Ethics approval was granted by the Northern Sydney Central Coast Health Human Research Ethics Committee, Sydney, NSW, Australia (protocol 1011-378M). Study participants were recruited from the Sydney-based practices of Otorhinolaryngologists, Nicholas Stow and Martin Forer, between August 2011 and July 2013. Patients requiring endoscopic sinus surgery on all paranasal sinuses for treatment of CRS were informed about the study and invited to participate by another researcher; if they indicated interest in participating, the surgeon obtained written informed consent. Diagnosis of CRS with or without NP was made based on the diagnostic criteria of the EPOS guidelines (Fokkens et al., 2012). Clinical data for each patient were collected, including demographics, SNOT-22 symptom scores (Kennedy et al., 2013), asthma status, and surgical history. Patients who had taken antibiotics or oral corticosteroids in the month prior to surgery were excluded from the study.

### Sample collection

General anesthesia was induced, and the nasal cavity was prepared by infiltration with a solution of 1% lignocaine and 1:100,000 adrenaline, and placement of pledgets soaked with 1:2000 adrenaline. Standard functional endoscopic sinus surgery, using microdebrider (Medtronic) and through-cutting instruments (Storz), was used to preserve mucosa while creating the largest possible sinus openings via natural ostia. As each sinus was opened, two sterile Copan Amies Transport swabs (Interpath Services, Melbourne, Victoria, Australia) were introduced into the sinus, one at a time, under endoscopic guidance, taking care to avoid contamination from other sites. One of these swabs was placed in the proprietary transport media provided with the swab and sent for culturing, while the other was placed in a sterile cryotube on dry ice for transportation, and stored at −80°C until DNA extraction. Each swab was taken from a different site within the same sinus, to minimize any effect on microbial abundance. Mucosal tissue was also collected from each diseased sinus using through-cutting instruments (Storz), and placed in a sterile cryotube prior to transportation, storage, and DNA extraction as per the swabs. A total of 22 swabs (two from the nostril, and then two from the middle meatus, maxillary sinus, ethmoid sinus, sphenoid sinus, and frontal sinus on each side) and eight tissue samples (one from each sinus on each side) were collected from each patient.

### Bacterial culture

Swabs were processed by Pacific Laboratory Medicine Services (St. Leonards, Sydney, Australia). Copan Amies Transport swabs without charcoal (Interpath Services, Melbourne, Victoria, Australia) were used. Each swab was transported to the abovementioned laboratory immediately and processed for culture and identification according to the following procedure. Swabs were inoculated onto horse blood, chocolate and Sabouraud agars, which were incubated aerobically, as well as agar designed to isolate Haemophilus species (horse blood agar with bacitracin, incubated in 5% CO_2_ atmosphere). Plates were incubated at 37°C and read at 24 and 48 h. Colonies were visually identified according to physical characteristics. Colonies suspected to be known pathogens such as *Streptococcus pneumoniae, S. aureus*, and *Haemophilus influenzae* were identified by standard methods. For other organisms, only those that grew as pure growth or predominant growth on the plates were sub-cultured for further characterization, speciation, and antibiotic susceptibility testing. Therefore, results were obtained and the organisms were classified as pathogenic or non-pathogenic. The method used excluded specific culture for anaerobes, a procedure that is not usually employed in the culture of specimens collected from a non-sterile site. Therefore, the organisms that were isolated are those for which this standard culture methodology is employed. As with most standard microbiology methods, the culture method that we used was tailored to isolate those organisms considered to be pathogens, rather than those considered to be non-pathogenic. It is acknowledged that this assumption may be erroneous, but it was anticipated that the molecular methods used would detect anaerobic and other bacteria that the standard culture would not detect.

### DNA extraction

DNA was extracted in batches from both the swab and mucosal tissue samples using the Allprep DNA/RNA Mini Kit (Qiagen). Samples were removed from storage at −80°C and transferred to Fastprep Lysing Matrix B tubes (MP Biomedicals) containing 600 μl RLT buffer using sterile instruments in a UV-decontaminated HEPA-filtered laminar flow hood. Tubes were placed in a Precellys 24 tissue homogenizer (Bertin Technologies) for 2 × 30 s at 6500 rpm with a 15 s pause, centrifuged at 12,000 rpm for 5 min, and the supernatants transferred to the Allprep DNA spin columns. The spin columns were processed according to the manufacturer's instructions, DNA was eluted using 100 μl EB buffer, and stored at −80°C until further processing.

### Library preparation and sequencing

The V4 region of the 16S gene was amplified from each of the DNA extracts using primers based on the design of Caporaso et al. (2012), modified to include an 8 bp barcode on both the forward and reverse primer rather than a single 12 bp barcode on the forward primer (Table 2). The amplification was carried out using two steps. In the first step, 28.5 μl of each DNA extract was used as the template for each 50 μl PCR, as it was found that maximizing the amount of template per reaction improved yield. This template underwent 10 cycles of PCR (15 s denaturation at 95°C; 30 s annealing at 50°C; 90 s extension at 72°C) with the modified Caporaso primers (500 nM), using different barcode combinations for each sample. Products were purified from the reactions using the Axyprep magnetic bead cleanup protocol (Axygen Biosciences). In the second step, the purified product of the first step was subjected to a further 25 cycles of PCR (15 s denaturation at 95°C; 30 s annealing at 55°C; 90 s extension at 72°C) using the enrichment primers (Table 2). The 25-cycle PCR protocol was selected after an initial trial of a 20-cycle protocol was found to produce a low yield. The PCR products underwent a final magnetic bead PCR cleanup using the Axyprep magnetic beads. All PCRs used the Taq Core PCR Kit (Qiagen) with dNTPs at 250 μM, and both forward and reverse primers at 0.5 μM each. Negative controls were included with each batch of PCRs.

**Table 2:** **Primers used for PCR amplification of V4 region of 16S gene**.

**Primer name**	**Primer sequence**
16S forward primer	AATGATACGGCGACCACCGAGATCTACAC(8bp_barcode)TATGGTAATTGTGTGCCAGCMGCCGCGGTAA
16S reverse primer	CAAGCAGAAGACGGCATACGAGAT(8bp_barcode)AGTCAGTCAGCCGGACTACHVGGGTWTCTAAT
Enrichment primer 1	AATGATACGGCGACCACCGA
Enrichment primer 2	CAAGCAGAAGACGGCATACGA

Double-stranded DNA concentrations of the purified PCR products were assayed using the Qubit dsDNA High Sensitivity Assay Kit (Invitrogen), and pooled to create a library with approximately equal concentrations of 16S amplicons from each sample. This library was sequenced on an Illumina MiSeq using the Reagent Kit V2 with 500 cycles (Illumina) and custom primers as described by Caporaso (Caporaso et al., 2012). The sequence dataset was demultiplexed and the read pairs merged using the Phylosift (Darling et al., 2014) and Flash (RRID:OMICS_01047; Magoc and Salzberg, 2011) software packages.

### Sequence analysis

The QIIME software package version 1.7 (RRID:OMICS_01521; Caporaso et al., 2010) was used to perform Operational Taxonomic Unit (OTU) picking and taxonomy assignment, and alpha and beta diversity analyses. Sequences were quality-filtered using the split_libraries.py script to exclude lengths shorter than 240 bp or longer than 260 bp, or with an average quality score less than 25. OTU picking and taxonomy assignments were performed using the pick_closed_reference_otus.py workflow script, which used the uclust method to cluster sequences against a version of the Greengenes reference database pre-clustered into OTUs at 97% similarity (version 8_13 obtained from the QIIME resources website http://qiime.org/home_static/dataFiles.html), and taxonomy was assigned based on this reference database. Singleton OTUs and OTUs for which the representative sequence failed alignment were removed as part of the workflow script.

Alpha diversity was analyzed using the alpha_rarefaction.py script to calculate observed counts of OTUs for each sample. Wilcoxon Rank Sum Tests were used to test for significant differences in alpha diversity between groups, implemented with the stats package in R.

Beta diversity was analyzed using the beta_diversity_through_plots.py workflow script with 2900 sequences per sample using the –e option to define depth of coverage for even sampling. The output of this command is matrix of weighted unifrac distances between samples (Lozupone et al., 2007) and PCoA plots. Different distributions of unifrac distances for various comparisons (e.g., all swabs within patients, all swabs between patients) were visualized using the make_distance_boxplots.py command in QIIME, and statistical significance between groups was assessed using Two-sample Kolmogorov-Smirnov tests, implemented in the stats package in R.

Differences in beta diversity associated with prior sinus surgery, or patient NP, or asthma status, were analyzed with a single PERMANOVA test, with prior sinus surgery, NP and asthma status as factors. Differences between sinus swabs and tissues within patients were tested using PERMANOVA, with swab or tissue status and patient as factors, and limiting permutations to within the patient variable using the strata parameter. Differences between nostril swabs and sinus swabs within patients were tested using PERMANOVA, with nostril or sinus swab status and patient as factors, and limiting permutations to within the patient variable using the strata parameter. All PERMANOVA tests were carried out with the adonis function in the Vegan package in R.

## Results

Samples were collected from 22 patients with CRS. The 22 swab and 8 tissue samples from each patient generated a collection of 418 samples for sequencing and 242 samples for culture. Despite repeated troubleshooting of PCR conditions, PCR amplicons were not detectable for any of the samples taken from patient 013 or patient 020, and all samples from patient 007 were lost prior to processing. These patients were excluded from further analysis, meaning that samples from 19 CRS patients were analyzed. A subset of samples was selected for sequencing, to determine whether sinus variability existed before processing numerous samples that could potentially provide identical results. 16S rRNA gene amplicons were not obtained for all samples; titration of internal positive controls of standard amounts of *E. coli* genomic DNA (detectable to 50 pg per 50 μl PCR) indicated that this was likely due to a low concentration of bacterial DNA in the samples relative to total DNA extracted which also contains host DNA. Of the 418 samples in the collection, amplification of the 16S rRNA gene was attempted for 179, and sequence data was obtained for 105 of those (Table 3). Negative controls included with each batch did not contain amplicons detectable by gel electrophoresis or Qubit dsDNA High Sensitivity Assay. Of the 19 patients for which 16S sequence was obtained, 14 were men and 5 women, and ages ranged from 18 to 69 (average age 45). SNOT-22 scores, as well as past sinus surgery and asthma status are shown in Table 4. Seven of the 19 patients were found to have NP on endoscopic examination, and these patients are identified in Table 4.

**Table 3:** **Sample collection and processing**.

**Patient ID**	**Left**									**Right**									
	**Frontal**		**Sphenoid**		**Ethmoid**		**Maxillary**		**Meatus**	**Nostril**	**Meatus**	**Maxillary**		**Ethmoid**		**Sphenoid**		**Frontal**	
	**Swab**	**Tissue**	**Swab**	**Tissue**	**Swab**	**Tissue**	**Swab**	**Tissue**	**Swab**	**Swab**	**Swab**	**Swab**	**Tissue**	**Swab**	**Tissue**	**Swab**	**Tissue**	**Swab**	**Tissue**
001	–	–	–	–	✔	–	**x**	✔	–	✔	–	✔	✔	✔	–	–	–	–	–
002	–	–	–	–	✔	–	✔	–	–	–	–	✔	–	**x**	–	–	–	–	–
003	–	–	–	–	✔	–	✔	✔	–	–	–	✔	**x**	✔	–	–	–	–	–
004	✔	–	✔	–	✔	–	**x**	✔	–	✔	–	✔	✔	✔	–	**x**	–	**x**	–
005	–	–	–	–	✔	–	✔	✔	–	–	–	✔	**x**	✔	–	–	–	–	–
006	✔	✔	✔	✔	–	–	**x**	–	–	✔	–	**x**	–	–	–	**x**	–	**x**	–
007	–	–	–	–	–	–	–	–	–	–	–	–	–	–	–	–	–	–	–
008	✔	✔	–	–	**x**	–	–	–	–	–	–	–	–	✔	✔	–	–	✔	✔
009	–	–	–	–	–	–	**x**	✔	–	–	–	✔	–	–	–	–	–	–	–
010	**x**	–	–	–	–	–	**x**	–	–	✔	–	**x**	–	–	–	–	–	✔	**x**
011	–	–	–	–	–	–	**x**	–	–	✔	–	✔	–	–	–	–	–	–	–
012	–	–	–	–	–	–	**x**	–	–	✔	–	✔	–	–	–	–	–	–	–
013	**x**	–	–	–	**x**	–	**x**	**x**	–	**x**	–	**x**	**x**	**x**	–	–	–	**x**	–
014	–	–	–	–	–	–	**x**	–	–	**x**	–	✔	–	–	–	–	–	–	–
015	–	–	–	–	–	–	✔	–	–	–	–	✔	–	–	–	–	–	–	–
016	–	–	–	–	–	–	✔	–	–	–	–	✔	–	–	–	–	–	–	–
017	**x**	–	✔	–	✔	✔	**x**	**x**	–	✔	–	✔	✔	**x**	**x**	**x**	–	**x**	–
018	✔	✔	✔	✔	✔	✔	✔	✔	✔	✔	✔	✔	**x**	✔	**x**	✔	✔	✔	**x**
019	✔	✔	✔	✔	**x**	✔	✔	**x**	✔	✔	✔	✔	✔	✔	✔	**x**	**x**	**x**	**x**
020	**x**	–	–	–	**x**	–	**x**	**x**	–	**x**	–	**x**	**x**	**x**	–	–	–	**x**	–
021	**x**	**x**	**x**	**x**	**x**	**x**	✔	✔	✔	**x**	✔	**x**	✔	**x**	**x**	**x**	**x**	**x**	**x**
022	✔	✔	✔	✔	✔	**x**	✔	**x**	✔	✔	✔	**x**	✔	✔	**x**	✔	**x**	✔	✔

Sequencing was successful for samples marked “✔,” PCR was unsuccessful for samples marked “x,” and PCR was not attempted for samples marked “–.”

**Table 4:** **Data summary for each patient from both swabs and tissue**.

**Patient ID**	**001**	**002**	**003**	**004**	**005**	**006**	**008**	**009**	**010**	**011**	**012**	**014**	**015**	**016**	**017**	**018**	**019**	**021**	**022**	**Mean**
SNOT-22	66	29	14	70	61	71	53	38	36	95	57	49	76	85	4	42	68	36	58	53
Past sinus surgery	Y	N	N	N	N	N	Y	N	N	N	N	N	Y	Y	Y	Y	N	N	N	32%
Asthma	Y	Y	N	N	Y	Y	N	N	Y	Y	N	N	Y	Y	Y	N	N	Y	N	53%
Nasal polyps	N	N	Y	N	N	Y	Y	N	Y	N	N	N	Y	Y	N	N	Y	N	N	37%
Number of samples	6	3	5	8	5	5	6	2	2	2	2	1	2	2	6	16	13	5	14	6
**AVERAGE PERCENT ABUNDANCE (STANDARD DEVIATION)**																				
*Actinomyces*	0(0)	0(0)	1(1)	0(1)	0(0)	0(0)	0(0)	0(0)	1(1)	0(0)	0(0)	0(0)	0(0)	16(16)	0(0)	0(0)	0(0)	0(0)	0(0)	1
*Corynebacterium*	14(7)	33(5)	25(22)	50(20)	34(11)	15(10)	30(19)	44(44)	76(1)	53(15)	22(1)	0(0)	2(1)	28(9)	43(10)	50(13)	74(14)	63(25)	42(9)	37
*Rothia*	0(0)	0(0)	7(4)	0(0)	0(0)	0(0)	0(0)	0(0)	0(0)	0(0)	0(0)	0(0)	0(0)	0(0)	0(0)	0(0)	2(2)	0(0)	0(0)	1
*Staphylococcus*	79(8)^*^	26(3)^*^	8(4)^*^	1(1)^*^	42(10)^*^	13(10)^*^	13(10)^*^	2(2)^*^	11(0)^*^	4(0)	2(1)	0(0)	0(0)	7(1)^*^	22(6)^*^	19(14)^*^	6(2)	6(2)^*^	23(3)^*^	15
*Alloiococcus*	0(0)	19(4)	3(4)	36(15)	0(0)	0(0)	0(0)	0(0)	0(0)	0(0)	20(8)	0(0)	0(0)	0(0)	0(0)	0(0)	0(0)	0(0)	0(0)	4
*Streptococcus*	0(0)	9(3)^*^	8(9)	2(5)	1(0)	5(5)	0(0)	0(0)	0(0)	0(0)	0(0)	0(0)	0(0)	23(21)	1(0)	0(0)	0(0)	0(0)	0(0)	3
*Anaerococcus*	2(2)	5(2)	15(10)	0(1)	2(2)	0(0)	1(1)	0(0)	2(0)	4(2)	0(0)	0(0)	0(0)	4(2)	14(5)	10(5)	10(7)	14(11)	11(5)	5
*Finegoldia*	0(0)	1(0)	5(3)	0(0)	0(0)	0(0)	1(2)	0(0)	1(0)	2(0)	0(0)	0(0)	0(0)	8(5)	1(1)	3(3)	1(1)	1(1)	4(2)	1
*Peptoniphilus*	0(0)	3(1)	7(3)	0(0)	0(0)	0(0)	1(1)	0(0)	1(0)	0(0)	0(0)	0(0)	0(0)	5(2)	6(7)	8(5)	0(0)	10(9)	9(4)	3
*Escherichia*	0(0)	0(0)	0(0)	0(0)	13(5)^*^	1(2)	17(11)	0(0)	1(1)	29(10)	0(0)	0(0)	0(0)	1(1)	0(0)	0(0)	0(0)	0(0)	0(1)	3
*Pantoea*	0(0)	0(0)	0(0)	0(0)	0(0)	2(4)	15(26)^*^	0(0)	0(0)	1(0)	0(0)	0(0)	0(0)	0(0)	0(0)	0(0)	1(1)	0(0)	0(0)	1
*Haemophilus*	1(2)	0(0)	3(2)	0(1)	0(0)	2(2)	0(1)	51(47)^*^	0(0)	0(0)	0(0)	97(0)^*^	96(2)^*^	1(1)	0(0)	0(0)	0(0)	0(0)	0(1)	13
*Moraxella*	0(0)	0(0)	0(0)	0(0)	0(0)	0(0)	0(0)	0(0)	0(0)	0(0)	56(10)^*^	0(0)	0(0)	0(0)	0(0)	0(0)	0(0)	0(0)	0(0)	3
*Pseudomonas*	0(0)	0(0)	1(2)	2(3)	1(2)	43(11)^*^	5(9)	0(0)	0(0)	0(0)	0(0)	0(0)	0(0)	1(0)	1(1)	1(1)	1(1)	0(0)	5(8)	3
Other (<5% per genus)	3(0)	4(0)	17(6)	7(5)	7(3)	18(6)	16(15)	1(0)	5(1)	5(1)	1(0)	3(0)	1(0)	6(0)	11(2)	8(2)	6(2)	6(2)	6(2)	7

Patient symptom scores (SNOT-22), past surgery, asthma, and NP status. Number of samples successfully sequenced for each patient. Average percentage sequence counts for each bacterial genus with a >5% sequence count in at least one patient. Asterisks (

* *) indicate genera that were identified by culturing as well. Shading represents the relative sequence count range (0%, 1−9%, 10−24%, 25−49%, 50−100%)*.

### Bacterial community variation between patients

A total of 6 160 342 sequences from 83 swab and 31 tissue samples (including six technical replicates) passed the quality filters and were assigned taxonomy. These sequences have been deposited in the NCBI SRA under accession number SRP064379. The sequencing depth per sample ranged from 2900 to ~31,000, thus sequences were rarefied to 2900 per sample for further analyses. The total number of sequences assigned to each sample can be seen in Table S2. There were 14 genera that made up greater than 5% of sequences for at least one patient; these, along with their average sequence abundance across all samples for each patient, are shown in Table 4. *Corynebacterium* was a significant component of the bacterial community for most of the patients, and accounted for the greatest proportion of sequences in 13 out of 19 patients. *Staphylococcus* was also found in most patients, with varying abundance. Other bacterial genera that were found in high abundance were: the obligate anaerobes *Finegoldia, Anaerococcus, Peptoniphilus*; the facultative anaerobes *Escherichia* and *Haemophilus*; and the aerobes, *Alloiococcus, Streptococcus*, and *Pseudomonas*. Cyanobacteria were identified in 17 of 19 patients, with a maximum of 2.4% sequence abundance (patient 004). The results shown in Table 4 demonstrate that bacterial community composition varied between patients, with different taxa dominating in different individuals. Variation of bacterial community composition associated with patient factors (past sinus surgery, asthma, and NP) was analyzed using PERMANOVA, which showed that these factors had statistically significant but very small effect sizes (prior surgery *R*^2^ = 0.02, *p* = 0.037, NP *R*^2^ = 0.06, *p* = 0.001; asthma *R*^2^ = 0.03, *p* = 0.004).

Genera identified by sequencing generally correlated well with the culturing results, for example: patients 014, and 015 (NP) had *H. influenzae* cultured, and had high sequence counts from sinus swabs of *Haemophilus*; *H. influenzae* was also cultured from patient 009 however high sequence counts were only observed in the tissue sample. Patient 012 had *Moraxella catarrhalis* cultured, and had high sequence counts of *Moraxella*. Patient 006 (NP) had *P. aeruginosa* identified by culture, and had high sequence counts of *Pseudomonas*. *Staphylococcus* species were sometimes cultured from sinuses with very low *Staphylococcus* sequence counts, perhaps because the culturing method was particularly sensitive to *Staphylococcus*, or because DNA extraction is less efficient for Gram-positive bacteria. There was only one case where a bacterial genus was identified by culture that was not detected by sequencing: *Enterobacter cloacae* was isolated from a patient 008 (NP) sample, but no *Enterobacter sp*. was identified by sequencing; however sequencing did identify *Pantoea agglomerans*, a closely-related Gram-negative rod. *S. aureus* was cultured from the sinuses of patients 001, 008 (NP), 018, and from the nasal swab (but not from the sinuses) of patient 004. Note that in most cases the sequences of the V4 region of the 16S gene were not sufficient to resolve identities to the species level.

### Variation of bacterial communities between sinuses

**Figure 1:**
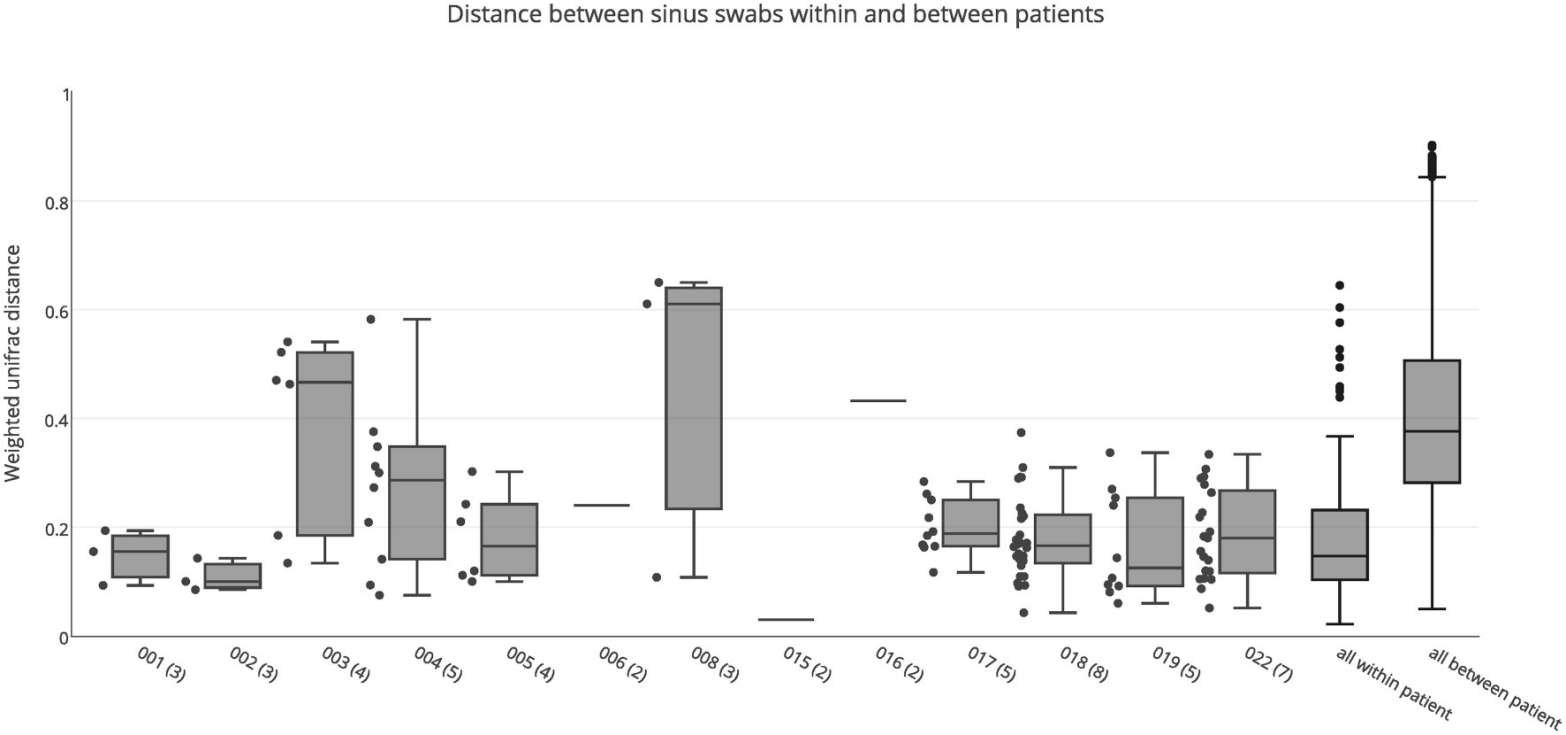
**Weighted unifrac distances between individual swab samples for each patient, as well as all intra-individual (all within patient) and inter-individual (all between patient) distances**. The number of samples for each patient is shown in brackets next to the patient ID. Boxes represent second and third quartiles; whiskers represent 1.5 times the interquartile range, and all individual data points are shown beside the boxes (where more than one data point is available per patient), except for the all within and all between patient groups.

Both weighted and unweighted unifrac distance matrices were generated to assess the variation between bacterial communities in the different sinuses of each patient, based on sinus swabs only. The unifrac distance is a value between 0 and 1, representing the proportion of phylogenetic branch length that is unique to one population being compared to another. A score of 0 indicates no unique branch length (i.e., communities are completely identical), while 1 indicates 100% unique branch length (communities are completely different). Unweighted unifrac values are based on the presence or absence of individual taxa in the tree, while the weighted unifrac distance is adjusted by the abundance of sequence counts that fall into each of the unique phylogenetic branch tips. Figure 1 shows a box plot of the weighted unifrac distances between swab samples taken from the different sinuses of each patient. A box plot of the unweighted distances is provided as Figure S1.

The median unweighted unifrac distances for each patient ranged from 0.47 to 0.69, indicating differences in community structure of between 47 and 69%. However, replicate PCRs and sequencing data from the same swabs also showed relatively high unweighted unifrac distances of 0.45–0.54. This is likely to be caused by the presence or absence of the many low abundance OTUs in each sample (see Figure S2), since all OTUs are given equal weight in the unweighted unifrac metric. Similarly high unweighted unifrac distances for technical replicates have previously been reported (Flores et al., 2012). The median weighted unifrac distances tended to be lower, ranging from 0.1 to 0.61, but were much higher than those obtained for replicate PCR samples (0.03–0.07). As such, we considered the weighted unifrac distances to be more suitable to detecting differences between samples for this analysis.

**Figure 2:**
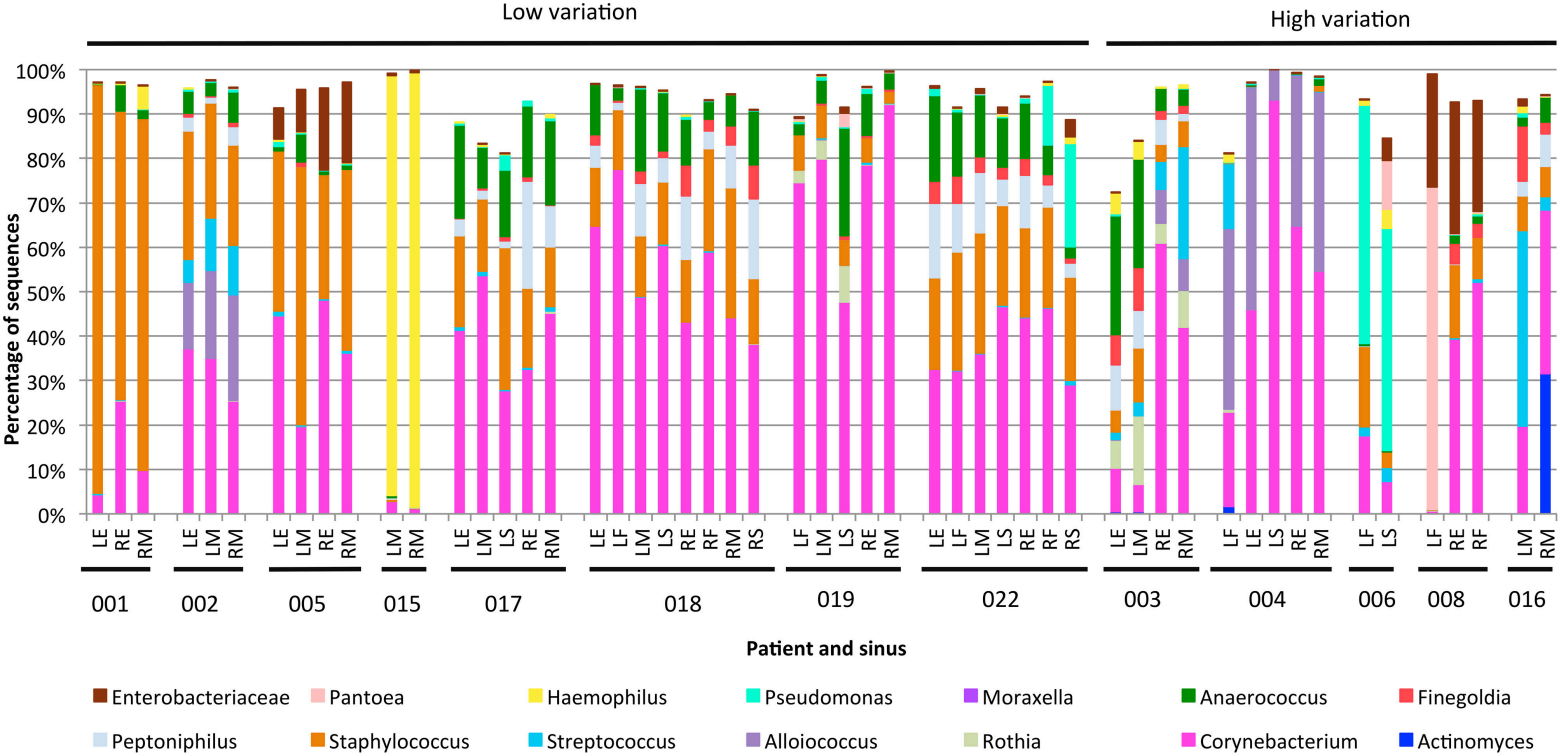
**Bar plots showing bacterial communities from individual sinus swabs of patients with low (<0.2) or high (>0.2) median between-sinus weighted unifrac distances**. Sinus locations are indicated by LE (left ethmoid), LF (left frontal), LM (left maxillary), LS (left sphenoid), RE (right ethmoid), RF (right frontal), RM (right maxillary), and RS (right sphenoid), with patient numbers indicated below. Only bacterial genera which represented at least 5% sequences in at least one sample were included.

The variability between sinuses within patients (median weighted unifrac value 0.15) was significantly less than variability across different patients (median weighted unifrac value 0.38) (Kolmogorov-Smirnov test, *p* < 0.001, *D* = 0.62). For some patients the weighted unifrac distances between sinuses were low (<0.2), indicating that the sampled sinuses had similar bacterial communities. For other patients the weighted unifrac distances between sinuses were higher (0.2–0.61), indicating that there was considerable variation between the bacterial communities in different sinuses of that patient. To further demonstrate the type and degree of variation seen, the sequence abundances of the most common bacterial genera are presented for individual sinuses of patients with low weighted unifrac distances (<0.2) and higher distances (>0.2) between sinuses (Figure 2). The sequence abundances for all samples are available as Table S1.

The exact frequency of within-patient, between-sinus variation cannot be determined due to the lack of sequence data for every sinus of every patient in the study, and lack of replicate swabs from each sinus within individuals. However, the sequencing results demonstrate that between-sinus variation does exist for some patients. Sinuses from patients with low median weighted unifrac values tend to be dominated by the same two to four genera, but with variations in the abundances of those genera (see Figure 2). Sinuses from patients with higher median weighted unifrac values tended to have more extreme differences in the abundances of one or more genera. For example, patient 003 had more anaerobic bacteria in the left sinuses, and *Streptococcus* and *Alloiococcus* in the right; patient 006 had more *Staphylococcus* in the left frontal sinus, and more *Pantoea* in the left sphenoid; patient 008 had a high sequence count of *Pantoea* in the left frontal sinus, and not in the right, which had much higher counts of *Corynebacterium*; patient 016 had a much higher sequence count of *Streptococcus* in the left maxillary than the right. Some of these sequencing results were corroborated by the culturing results, for example patient 008 had a pure culture of a bacteria identified as *E. cloacae* from the left frontal sinus only, which correlates with the high sequence count in that sinus for an OTU identified as *P. agglomerans* which is likely to be the same (see above).

### Variation of bacterial communities between swab and tissue samples

**Figure 3:**
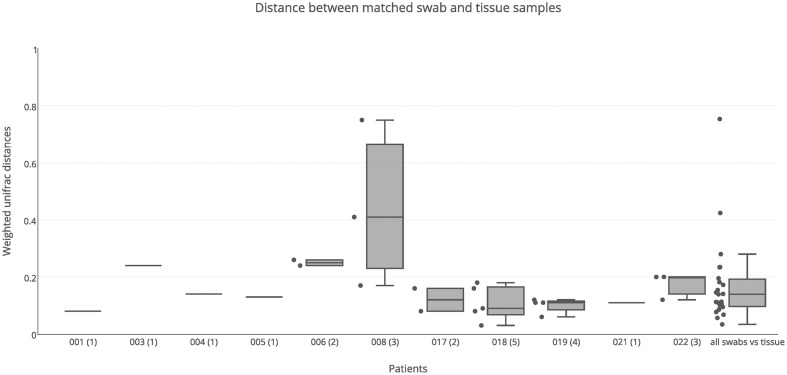
**Weighted unifrac distances between swab and tissue samples within patients**. Distances were plotted for matched swab and tissue samples (23 pairs) for each patient separately, and all together, The number of sinuses with sequence data for both swab and tissue samples is shown in brackets next to the patient ID. Boxes represent second and third quartiles; whiskers represent 1.5 times the interquartile range, and all individual data points are shown beside the boxes (where more than one data point is available per patient).

Both swab and tissue samples from the same sinus of certain patients were sequenced, to determine whether sampling method influences results; there were 23 paired samples in total. PERMANOVA analysis indicated that swabs and tissue samples were significantly different when taking inter-individual variability into account, however the type of sample only accounted for a small amount of the variation observed (*R* = 0.02, *p* = 0.028). The results obtained from swab samples were generally very similar to those obtained from tissue samples, with a median unifrac distance of 0.14 (Figure 3). For the majority of patients, weighted unifrac distances between swab and tissue samples of the same sinus were less than 0.2 (indicating >80% shared community structure). Patients 003 and 006 showed slightly higher unifrac distances between tissue and swabs from the same sinus (0.24–0.26) while patient 008 had very variable results (0.17–0.75). The swab of the right ethmoid sinus from patient 008 (0.75 weighted unifrac value in comparison with tissue) had high sequence counts of *Corynebacterium* (39%) and *Escherichia* (30%), while the tissue sample had respective counts of 7 and 1%, and a high count of *Pseudomonas* (24%). The swab of the left frontal sinus (0.41 weighted unifrac value in comparison with tissue) had high sequence counts of *P. agglomerans* (73%), while the tissue sample only had a low count for *P. agglomerans* (3%) and a high count of *Corynebacterium* (39%), and *Staphylococcus* (32%). Distances between matched tissue and swab samples was not significantly different to distances between sinuses from within individuals (Kolmogorov-Smirnov test, *p* = 0.87, *D* = 0.13), indicating that while tissue and swab samples yield some differences in microbial community profiles, these differences are no larger or smaller than the variations observed between different sinuses within an individual.

### Variation of bacterial communities between nostril and sinus swab samples

**Figure 4:**
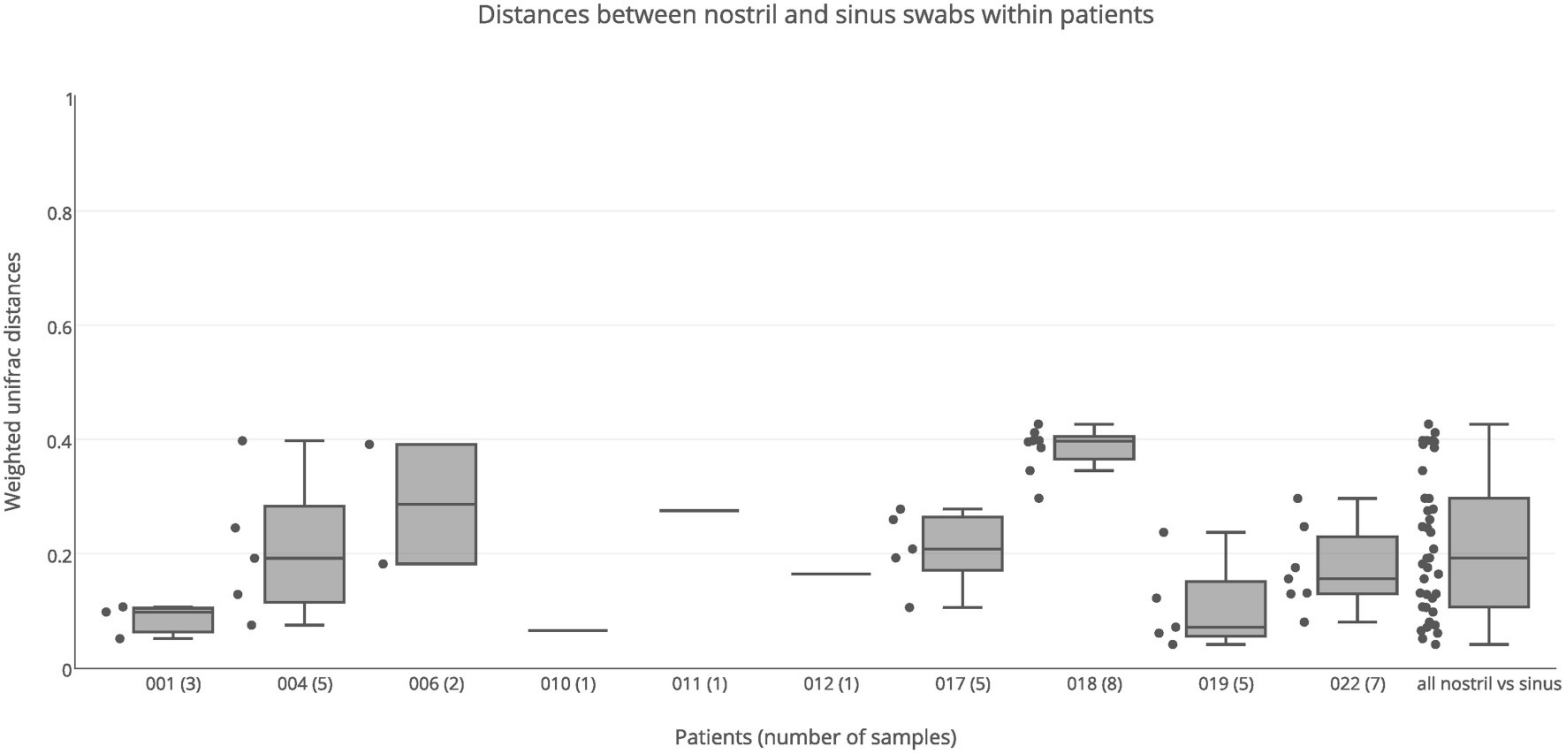
**Weighted unifrac distances between nostril swab samples and sinus swabs within patients**. Distances are plotted for each patient separately, and all together. The number of samples compared is shown in brackets next to the patient ID. Boxes represent second and third quartiles; whiskers represent 1.5 times the interquartile range, and all individual data points are shown beside the boxes (where more than one data point is available per patient).

Nostril swab samples were compared with sinus swab samples from the same patient, to determine whether a nostril sample is a reliable surrogate for sinus samples. PERMANOVA analysis found no significant difference between nostril and sinus samples when taking inter-individual variation into account (*R* = 0.02, *p* = 0.253). When broken down into individual patients, some had similar results for swab samples taken from their nostril and sinuses, while other patients had very different results between nasal and sinus samples (Figure 4). For example the nostril of patient 001 had a very similar *Staphylococcus*-predominant bacterial community in the nostril, ethmoid, and maxillary sinuses, with weighted unifrac distances all less than 0.10, while patient 018 had a *Staphylococcus*-predominant bacterial community in the nostril, but more *Corynebacterium* and anaerobe-dominated bacterial communities in the maxillary, ethmoid, sphenoid, and frontal sinuses, with a median weighted unifrac distance of 0.40. Several patients had bacterial genera with sequence counts >5% in their sinus samples that were in very low abundance in the nostril swab; for example the left frontal sinus of patient 004 had 14.6% *Streptococcus* and 13.5% *Veillonella* sequences, but the nostril sample had <0.01% of these sequences. Patient 006 had 10.9% *Pantonea* sequences in the left sphenoid sinus, but none in the nostril swab. In patient 018, *Anaerococcus* sequences account for more than 5% of sequences in all but one sinus (range 3.9–18.5%) but only account for 0.6% of sequences in the nostril swab. Distances between nostril and sinus swabs within an individual were not significantly different to distances between matched tissue and swab samples (Kolmogorov-Smirnov test, *p* = 0.15, *D* = 0.29) and while the median was slightly higher than for distances observed between sinuses within an individual, this difference did not reach significance (Kolmogorov-Smirnov test, *p* = 0.06, *D* = 0.22).

### Differences in alpha diversity between sample groups

No significant differences in alpha diversity were detected based on sample type (tissue or swab, Wilcoxon rank sum test, *p* = 0.19), location (nostril or sinus swab, Wilcoxon rank sum test *p* = 0.17), or patient factors (Wilcoxon rank sum test, asthma, *p* = 0.37, prior surgery *p* = 0.44, nasal polyps *p* = 0.09).

## Discussion

CRS can be a debilitating condition that is recalcitrant to treatment. Bacterial colonization of the sinuses is likely to play an important role in the pathogenesis and perpetuation of the disease; however different studies have yielded contrasting results with respect to which bacterial taxa are characteristic of the disease (Stephenson et al., 2010; Stressmann et al., 2011; Abreu et al., 2012; Feazel et al., 2012; Aurora et al., 2013; Boase et al., 2013). We observed bacterial communities dominated by different taxa in CRS patients; for example some have sinuses colonized primarily with *Haemophilus*, while others are dominated by *Corynebacterium* and *Staphylococcus*, or *Pseudomonas*. Some patients' sinuses contain anaerobic bacteria such as *Anaerococcus, Finegoldia*, and *Peptoniphilus*, while these were absent from others. Indeed, our results have shown, for the first time, that it is possible for a patient to simultaneously have different bacterial communities in different sinuses, pointing to distinct, localized microbiomes within the same patient. Understanding this variation in the sinus microbiome could prove critical to the appropriate selection of treatments for CRS in the future.

It was surprising that PCR amplification of the 16S gene proved difficult for some samples, particularly in the case of tissue samples. Adequate concentrations of DNA were extracted from all samples, but experiments using titration of internal positive controls demonstrated that for many samples the relative concentration of bacterial 16S DNA was very low. Alternative DNA extraction protocols were trialed without improvement of PCR amplicon yeild. Maximizing the concentration of DNA template in the PCRs improved the overall yield of 16S amplicon, but was not enough to obtain sufficient product from all samples. Other researchers have reported similar issues, for example Abreu et al. used a similar DNA extraction protocol and were unable to generate sufficient 16S amplicon for six of their 20 patients (Abreu et al., 2012). Future research projects might benefit from further optimization of DNA extraction methods or PCR primer design, although it should also be acknowledged that failure to detect a bacterial 16S amplicon does not rule out the presence of viruses, archaea, or other biological processes which may contribute to rhinosinusitis.

The weighted unifrac distances between samples within patients (Figure 1) demonstrate that at least some CRS patients have substantial variation of bacterial communities between sinuses, although it is significantly smaller than the variation observed between different individuals. While this variation was related to abundance rather than the presence or absence of dominant community members, some of these variations were large: for example *Corynebacterium* sequences dominating the right sinuses of patient 003 (60.7 and 41.7% of all sequences), while the left sinuses had much smaller abundances (9.8 and 6.2%) and were dominated by the anaerobic bacteria *Anaerococcus, Finegoldia*, and *Peptinophillus* (see Figure 2). This within-patient heterogeneity could have important implications for our understanding of the pathogenesis and pathophysiology of CRS. It is possible that this is also the case in non-CRS individuals, and future work will include sampling from a control cohort to compare microbial communities in sinuses with and without disease. Sampling from a single location, as previous researchers have done, may give an incomplete picture of the bacterial diversity in the sinuses. Although this finding may seem counterintuitive, considering the continuity of the sinus mucosa through the different sinuses, it is not difficult to imagine how the unidirectional flow generated by the cilia of the mucosa, along with the reduced patency of the ostia in CRS, could set up isolated environments with divergence of bacterial communities. Heterogeneity of microbial communities at different locations within the nostril cavity has previously been shown (Yan et al., 2013; Biswas et al., 2015), demonstrating that different microenvironments within an anatomical structure can be associated with differences in the associated microbial community. In addition, the culture results, although less sensitive to variations in community structure, were able to provide independent verification that within-patient, between-sinus variation does exist.

Sinuses were sampled using both swabs and tissue biopsies. No significant differences were detected in alpha diversity (number of OTUs detected) between swab and tissue samples, and while significant differences were detected in community composition and structure between swab and tissue samples, the amount of variation explained was very small (*R* = 0.02, *p* = 0.028), indicating that the type of sample collected (i.e., tissue or swab) has a very small effect on the results obtained. In most cases, both types of sampling produced similar 16S sequence data, consistent with a recent comparison of the two sampling methods (Bassiouni et al., 2015). There were two notable exceptions to this, both from different sinuses of the same patient. One sinus had high counts of *P. agglomerans* from the swab but not the tissue; this is an environmental organism that may be isolated from surfaces of plants and non-sterile sites in humans. It is rarely pathogenic in immunocompetent individuals, except in association with plant penetrating injury (Cruz et al., 2007). Another sinus had high counts of *Corynebacterium* and *Enterobacteriaceae* from the swab, but not the tissue. This discrepancy could be because the swabs are picking up more planktonic bacteria that are abundant in the mucus, while the tissue samples have greater numbers of intracellular bacteria or bacteria associated with biofilms. Whether future CRS research projects should collect both swab and tissue samples would depend on the focus of the research, as collecting and processing both is time consuming and costly, and in most cases the results are likely to be similar.

Swabbing the nostril is much easier than sampling the sinuses, as it does not require surgery. However, the microbiome of the nostril is generally considered to be distinct from the sinuses (Feazel et al., 2012). One objective of this project was to test how well the microbiome of the nostril represented the microbiome of the sinuses. No significant differences were found for either alpha diversity or beta diversity between nostril and sinus samples. The median distance between nostril and sinus samples from within an individual was slightly higher than that found between sinuses within an individual, and although this result did not reach significance, it was very close to the significance cutoff (*p* = 0.06). Weighted unifrac distances between the nostril and sinus samples of each patient showed that for some patients, such as patient 001, the nostril is quite a good representative of the sinuses, while for others, such as patient 018, the nostril microbiome has a very different bacterial composition to the sinuses. Considering the degree of variation that can be present in the different sinuses, it is not surprising that a single nostril swab will not always be representative of the sinus communities. Therefore, sampling of the nostril is not necessarily a reliable surrogate for sampling of the sinuses.

This research provides the first evidence that bacterial communities can vary between the sinuses of patients with CRS. This is important for directing the sampling methods of future research into the disease, as the current practice of collecting a swab or tissue from a single sinus may not provide representative data. This finding may also have implications for disease management, as these data show that pathogenic organisms could be missed when only a single sinus is sampled. The data presented here suggest that variation of the microbiome between different sinuses of people with CRS is likely to be at least 25%, and may be considerably higher. The biological significance of this variation is not yet know, but a better understanding of sinus to sinus variation within patients (along with more standardized methods for sample collection and processing) will likely lead to improved knowledge of the pathogenesis of CRS. Another avenue for future research would be to investigate whether CRS patients with variation of bacterial communities between sinuses have differing degrees of inflammation or disease state associated with particular bacterial species. The fact that different sinuses of an individual CRS patient can harbor different pathogens should allow observations of the effect of certain bacterial species on the host with fewer confounding host-dependent factors.

## Funding

Funding for this study was provided by Medtronic, and the University of Technology Sydney.

### Conflict of interest statement

The authors declare that the research was conducted in the absence of any commercial or financial relationships that could be construed as a potential conflict of interest. Seed funding for this project was provided by Medtronic, a surgical equipment company. Medtronic had no access or control over the study design, data analysis, or reporting of the results.

## Supplementary material

Figure S1 **Unweighted unifrac distances between individual swab samples for each patient**. The number of samples for each patient is shown in brackets next to the patient ID. Boxes represent second and third quartiles; whiskers represent 1.5 times the interquartile range, and all individual data points are shown beside the boxes (where more than one data point is available per patient).

Figure S2 **Rank abundance curve of all sinus samples sequenced**. This shows that the top 10 abundant OTUs generally account for more than 99% of the sequences per sample, while the remaining several 100 OTUs per sample account for less than 1% of the sequences.

Table S1 **Relative sequence abundances for all genera and samples in the study**.

Table S2 **Sequence coverage per sample for swab and tissue samples**.
