# Supplementary figures and images for: Bacterial Communities Vary between Sinuses in Chronic Rhinosinusitis Patients
Source: Front Microbiol. 2016 Jan 22;6:1532. doi: 10.3389/fmicb.2015.01532 (PMC4722142; doi:10.3389/fmicb.2015.01532)

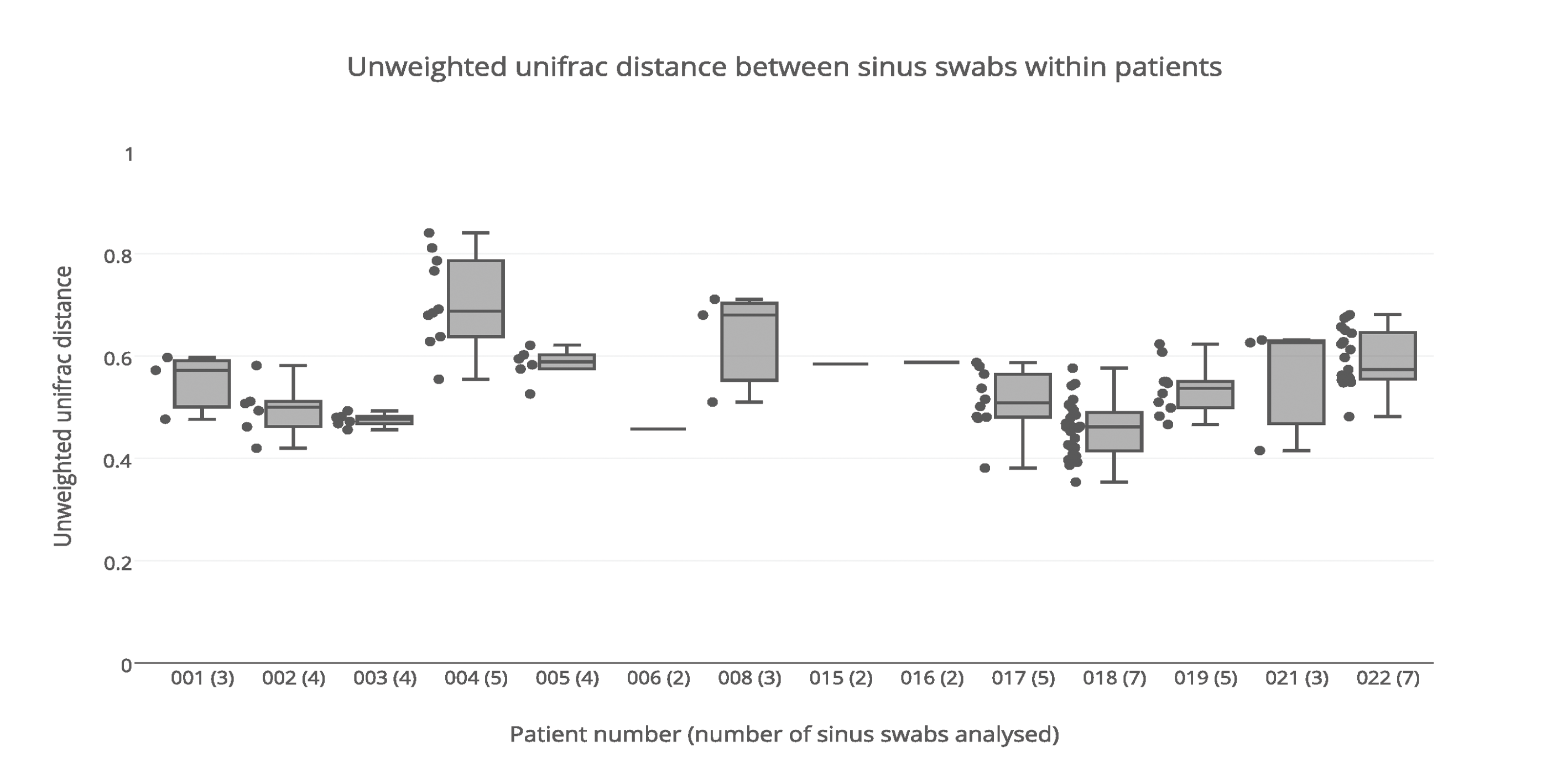

Supplement: Figure S1 — Unweighted unifrac distances between individual swab samples for each patient. The number of samples for each patient is shown in brackets next to the patient ID. Boxes represent second and third quartiles; whiskers represent 1.5 times the interquartile range, and all individual data points are shown beside the boxes (where more than one data point is available per patient). [file Image1.TIF]

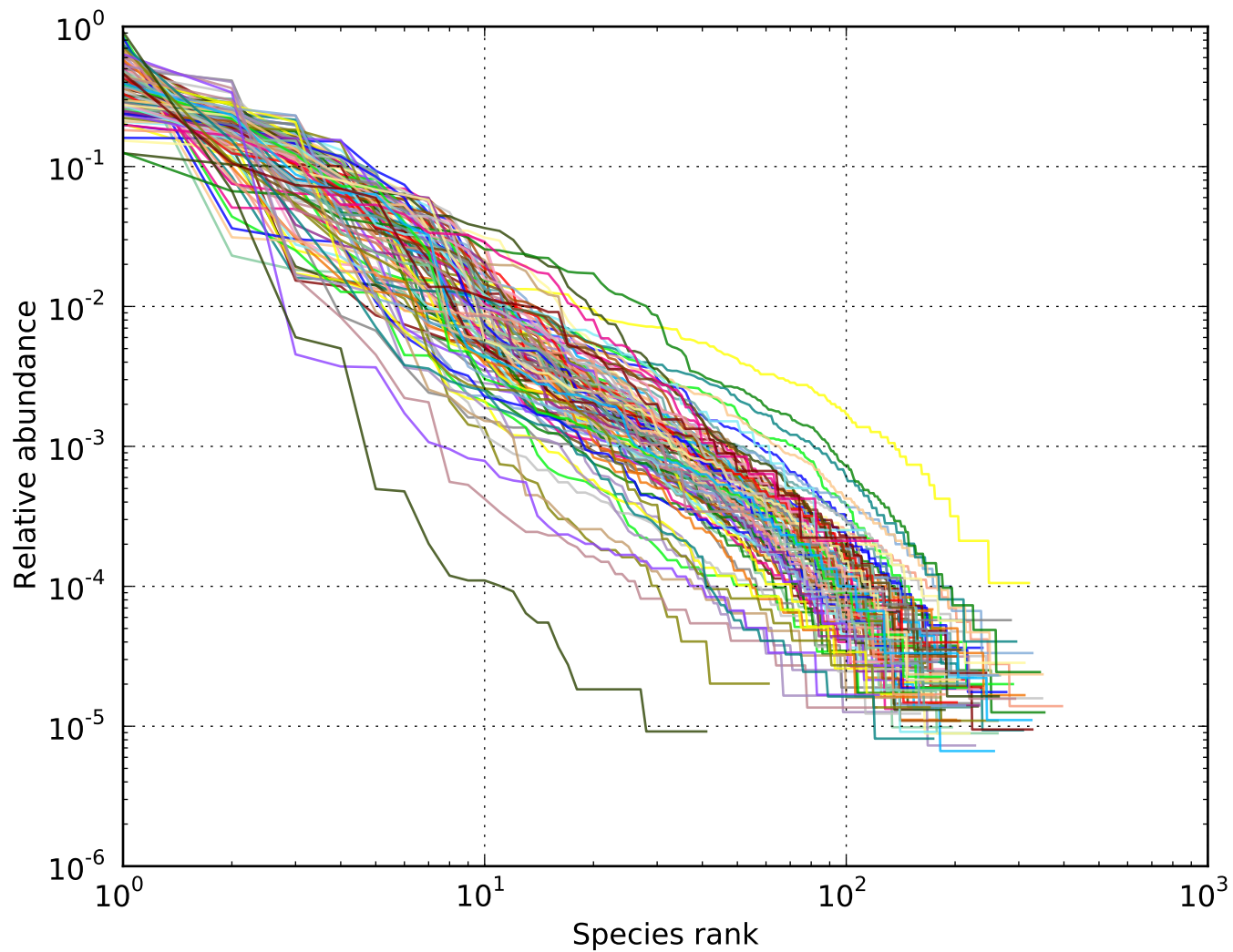

Supplement: Figure S2 — Rank abundance curve of all sinus samples sequenced. This shows that the top 10 abundant OTUs generally account for more than 99% of the sequences per sample, while the remaining several 100 OTUs per sample account for less than 1% of the sequences. [file Image2.PDF]
